# Evaluation of neutrophil gelatinase-associated lipocalin (NGAL), matrix metalloproteinase-9 (MMP-9) and their complex MMP-9/NGAL in sera and urine of patients with kidney tumors

**DOI:** 10.3892/ol.2013.1252

**Published:** 2013-03-13

**Authors:** ANGELINA DI CARLO

**Affiliations:** Department of Medico-Surgical Sciences and Biotechnologies, ‘Sapienza’ University of Rome, I-Rome 00161, Italy

**Keywords:** neutrophil gelatinase-associated lipocalin, matrix metalloproteinase-9, MMP-9/lipocalin complex, oncocytoma, clear cell renal cell carcinoma, serum, urine

## Abstract

Recent evidence suggests that neutrophil gelatinase-associated lipocalin (NGAL) is required for the development and/or progression of benign and malignant disease, and is overexpressed in several types of tumor. Matrix metalloproteinase-9 (MMP-9), by degrading components of the extracellular matrix and thus promoting the release of growth factors, is important in tumor growth and tumorigenicity. NGAL protects MMP-9 from proteolytic degradation and enhances its enzymatic activities by binding and forming the MMP-9/NGAL complex. Therefore, NGAL, MMP-9 and their complex MMP-9/NGAL have been proposed as soluble biomarkers for numerous malignancies. In the present study, we measured the concentration of these molecules in sera and urine of patients with kidney disease using ELISA. Of these patients, 16 had clear cell renal cell carcinoma (ccRCC) and 4 had oncocytoma. Sera and urine samples of 53 healthy patients were used as controls. In sera, MMP-9 was enhanced in ccRCC patients compared with oncocytoma patients. In urine, the most abundant molecule was NGAL and its mean value was higher in cancer patients. However, there was a broad overlap of the data and we did not identify any correlation with disease type, stage or grade. Therefore, these molecules may not be useful as biomarkers for predicting kidney carcinoma.

## Introduction

Lipocalin 2, also known as neutrophil gelatinase-associated lipocalin (NGAL), is a prominent member of the lipocalin family. The lipocalins constitute a large group of small, predominantly extracellular proteins previously regarded as obscure transporters of hydrophobic ligands (1,2 and refs. therein). In humans, NGAL was originally identified as a 25-kDa protein covalently linked to matrix metalloproteinase-9 (MMP-9) in human neutrophils (3), which normally provide the main cellular source of circulating NGAL. MMP-9, by degrading components of the extracellular matrix and thus promoting the release of growth factors, is important in tumor growth and tumorigenicity (4,5). By forming the MMP-9/NGAL complex, NGAL protects MMP-9 from proteolytic degradation, increasing the enzymatic activity of MMP-9 and subsequently enhancing tumoral invasiveness and diffusion (6).

NGAL expression has been studied in several normal tissues where it funtions to modulate oxidative stress and to provide protection against bacterial infection. Its expression is altered in several benign conditions, including inflammatory, ischemic and metabolic disorders. With regard to the kidney, NGAL is expressed in the epithelial cells and is involved in kidney development, where it has been demonstrated to regulate epithelial morphogenesis. Persistent high levels of NGAL lead to the development of proliferative renal lesions and chronic kidney disease via an epidermal growth factor receptor-dependent mechanism (7). Recent data have demonstrated that increased levels of NGAL are present in chronic kidney disease and acute kidney damage (8,9). Furthermore, it is overexpressed in numerous tumor types, including breast, thyroid, colorectal, gastric and pancreatic cancer (8). Observations in animal models and human subjects suggest that NGAL is required for the development and/or progression of benign and malignant disease, and its expression is associated with invasive cancer progression. Several studies have investigated the level of circulating NGAL in the blood as a potential marker for the detection and prognostication of solid tumor and hematological malignancies (8,10–13). However, it is likely that the level of NGAL is largely neoplasia-specific and influenced by tumor type.

In the present study, we measured serum and urinary levels of NGAL, MMP-9 and MMP-9/NGAL complex in patients with oncocytoma and clear cell renal cell carcinoma (ccRCC) in order to verify whether these molecules may offer a potential non-invasive biomarker to provide useful clinical information for kidney carcinoma.

## Materials and methods

### Patients

Patients were selected for the study and samples of their peripheral venous blood and first morning urine were collected prior to surgical or other therapeutic intervention. Specimens were obtained from patients who had undergone surgical procedure. Diagnosis of the tumor type was performed by usual clinical laboratory criteria and confirmed by histopathological observations. The age of patients ranged between 40 and 73 years (mean ± SD, 59.2±9.7) and in total, there were 11 males and 9 females. The tumors were classified by grade and stage according to the pTNM classification (14). All patients provided written informed consent. The study was approved by the local ethics committee. Normal, healthy laboratory volunteers provided their permission verbally. The healthy volunteers had no concomitant illnesses, including no signs of infection, gastrointestinal hepatic or renal disease. The values of the basic laboratory parameters of these participants were within the reference limits.

### Serum

Peripheral venous blood samples were collected in vacutainers, allowed to clot for 30 min at room temperature and centrifuged at 1,600 g for 10 min at 4°C. The samples were then divided into aliquots and stored at −20°C until used. Each aliquot was used only once in order to prevent enzyme activation due to freeze-thawing processes.

### Urine sample preparation

Prior to analysis, urine samples were examined using the Multistix Combur test (Roche Diagnostics GmbH, Mannheim, Germany). Urine samples which tested positive for leukocytes were excluded due to confounding leukocytic gelatinases. Microscopic hematuria present in the majority of cancer samples was not quantified, however, excessively hematuric samples were excluded. Samples were frozen immediately following collection and stored at −20°C until assay. The samples were thawed and an aliquot of each sample (15 ml) was centrifuged at 1,000 × g for 10 min at 4°C. Supernatant was collected and used to determine NGAL, MMP-9 and MMP-9/NGAL by immunoassay.

### Measurement of NGAL, MMP-9 and MMP-9/NGAL

NGAL and the MMP-9/NGAL complex levels were determined by a solid-phase immunoassay using commercial kits obtained from R&D Systems (Minneapolis, MN, USA). The NGAL assay used two monoclonal antibodies specific for two different epitopes of lipocalin 2. The MMP-9/NGAL complex assay used monoclonal antibodies raised against recombinant human MMP-9 and recombinant human NGAL, and is subsequently not capable of detecting recombinant human MMP-9 or NGAL in their free forms. MMP-9 was detected by immunoassay (ELISA) with a commercial kit obtained from GE Healthcare (Buckinghamshire, UK). This assay is based on a two-site sandwich format using two antibodies directed against different epitopes of the molecule; the assay recognises the precursor of MMP-9 (proMMP-9) and that complexed with TIMP-1 (proMMP-9/TIMP-1 complex). All analyses were performed according to the manufacturer's instructions.

### Statistical analysis

All statistical analyses were performed with the statistical computing environment R (version 2.12.1; R Foundation for Statistical Computing, Vienna, Austria). Results are summarized as the mean ± standard deviation (SD). Fisher's exact test was performed. P<0.05 was considered to indicate a statistically significant difference.

## Results

### Patients

During a 1-year period, a total of 20 patients with kidney disease were evaluated. Of these patients, 4 had oncocytoma and 16 had ccRCC. For each of the patients, a venous blood sample was collected, and for four of the patients with oncocytoma and 9 patients with ccRCC, first morning urine samples were obtained. All three molecules, including NGAL, MMP-9 and their complex MMP-9/NGAL were measured in serum and in urine samples. Since normal values for these molecules were unavailable, we measured these molecules in sera and in the urine of 53 healthy subjects, which was considered as the control group.

### Serum samples

**Figure 1:**
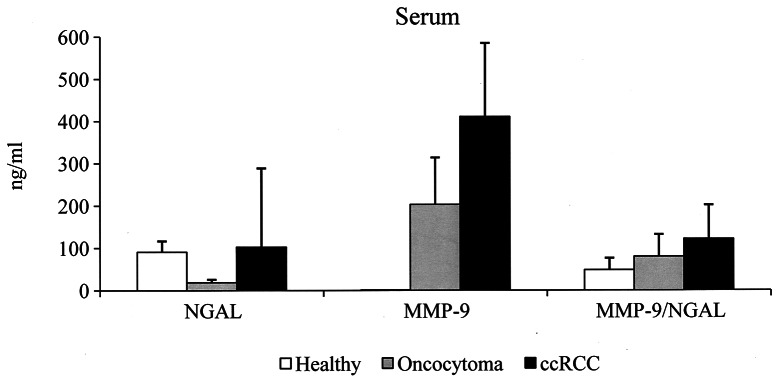
Mean + standard deviation of serum NGAL, MMP-9 and MMP-9/NGAL complex expression. NGAL, neutrophil gelatinase-associated lipocalin; MMP-9, matrix metalloproteinase 9; ccRCC, clear cell renal cell carcinoma.

In sera of the control group, NGAL was detected in all samples with a value ranging between 35 and 153 ng/ml (91±26). MMP-9 was undetectable at levels equal to or below the sensitivity of the assay. MMP-9/NGAL complex was detected in all specimens and ranged between 9 and 145 ng/ml (48±28). We established the cut-off value by calculating the mean + 2SD and the following cut-off values were considered; 143 ng/ml for NGAL and 104 ng/ml for MMP-9/NGAL. Samples with a value higher than that of the cut-off were considered positive. The data obtained in sera from patients with oncocytoma and ccRCC are shown in Tables I and II, respectively. In oncocytoma, NGAL values were negative in all specimens analyzed, while in ccRCC, values were positive in 2/16 (12.5%) of specimens (range, 6–750; 103±186). MMP-9 was detected in all specimens analyzed and with values of 203±111 (range, 82–327) and 411±174 (range, 168–730) observed in oncocytoma and ccRCC patients, respectively. Since serum MMP-9 was undetectable in all healthy subjects, we considered all pathological specimens positive as all samples possessed serum MMP-9 values higher than the sensitivity of the assay (assay sensitivity has been calculated by two standard deviations above the zero dose binding of 80 determinations and was 0.8 ng/ml). MMP-9/NGAL complex in oncocytoma was positive in 25% (1/4) of samples (range, 18–145; 80±52), and was positive in 44% (7/16) of ccRCC specimens (range, 14–306; 122±80). Considering the average value of each molecule, we observed that the values are higher in sera from ccRCC patients compared with those of oncocytoma patients. In addition, the serum MMP-9 level is 2-fold higher in ccRCC compared with oncocytoma specimens (Fig. 1).

### Urine samples

**Figure 2:**
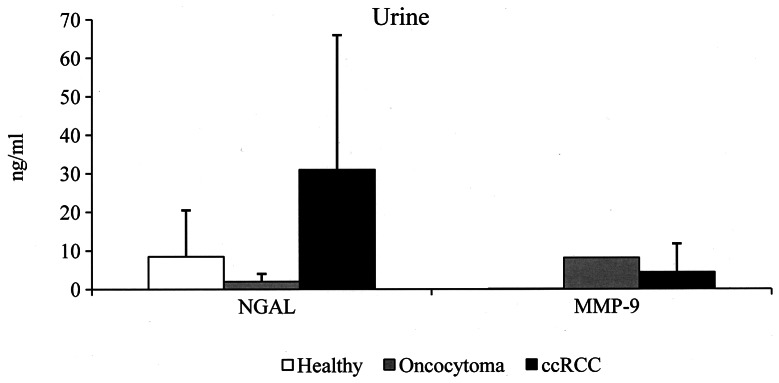
Mean + standard deviation of urinary NGAL and MMP-9 expression. NGAL, neutrophil gelatinase-associated lipocalin; MMP-9, matrix metalloproteinase 9; ccRCC, clear cell renal cell carcinoma.

With regard to urine specimens, NGAL was detected in all urine samples of the control group with a value ranging between 0.1 and 52 ng/ml (8.5±12), with 32.5 ng/ml being considered as the cut-off value. By contrast, no cut-off value for urinary MMP-9 and urinary MMP-9/NGAL complex was established since MMP-9 was undetectable in all specimens analyzed and the MMP-9/NGAL complex was detectable in only 10% of urine samples from normal healthy subjects. In oncocytoma, urinary NGAL values were negative in all specimens analyzed, whereas 44% (4/9) of ccRCC specimens were positive (range, 0.9–116; 31±35.5). Urinary MMP-9 was detected in only one oncocytoma specimen with a value of 8.13 ng/ml, and in 67% (6/9) of ccRCC patients (range, 0.55–22.8; 4.38±7.4). MMP-9/NGAL complex was undetectable in all urine samples analyzed from oncocytoma and ccRCC patients (Tables III and IV). The average value of NGAL and MMP-9 of the positive urine specimens is shown in Fig. 2. It is evident that the most abundant substance in ccRCC patients is NGAL and that the mean value is 3.6-fold higher than that detected in the urine samples of the control group. Secondly, the mean value of urinary MMP-9 is less than that of sera. Finally, specimens P19 (T3N0M1, G3) and P20 (T3bN0M1, G3) demonstrated metastasis of the bone. We demonstrated that serum and urine values of NGAL in these patients were not increased compared with the mean values of healthy subjects.

## Discussion

Renal cancer is generally silent and frequently fatal. The majority of kidney tumors are discovered co-incidentally during abdominal imaging performed for unrelated diagnostic reasons. Currently, no diagnostic modality for the early detection of renal cancer exists, other than incidental radiological discovery. In addition, no method capable of monitoring recurrence currently exists. Thus, there is a great interest in identifying ‘biomarkers’ that are capable of improving this situation. Several tumor markers have been examined previously, however, there are no definitive biomarkers available for such purposes (15). Among protein markers, MMP-2 and MMP-9 have been investigated with variable results (16 and refs. therein). By gelatin zymography, we have previously demonstrated that the most abundant serum lytic activity was at 92 kDa (MMP-9) and that MMP-9 activity was slightly enhanced in sera from ccRCC patients compared with oncocytoma patients. In the present study, we obtained identical results using ELISA. However, there was a broad overlap of the data and we identified no correlation to the type of carcinoma, pathological TNM stage or histological grading.

NGAL is a biomarker of tubular injury, is expressed in several histotypes of renal tumors and its high expression is associated with a higher histological grade of ccRCC and papillary RCC, whereas oncocytoma and urothelial carcinoma exhibit lower expression levels (17). By immunohistochemistry, Perrin *et al* showed that NGAL was expressed by neutrophils infiltrating ccRCC and that the density of NGAL-expressing neutrophils was associated with poor outcomes (18). Furthermore, they reported that high NGAL concentrations in serum were also associated with shorter progression-free survival (18). In patients treated with sunitinib, Porta *et al* demonstrated that serum levels of NGAL were significant predictors of progression-free survival (19). NGAL appears to protect MMP-9 from autodegradation, increasing its activity by binding and forming MMP-9/NGAL complexes. Tumor cells excrete elevated levels of NGAL resulting in an increase of the local concentration of MMP-9, which is capable of affecting various aspects of tumor progression. High concentrations of MMP-9/NGAL complex in serum have been associated with a shorter progression-free survival and poor overall survival (18). The results outlined in the present study indicate that the mean values of serum NGAL and MMP-9/NGAL complex are higher in ccRCC patients compared with oncocytoma patients. However, there was a broad overlap of the data and we observed no correlation with kidney disease severity.

Following localized tumors or monitoring drug-based therapy results by analyzing tumor-specific markers in the available excretory product of the kidney is highly desirable. However, to the best of our knowledge, there is only limited literature available with regard to urine markers for RCC. Concerning NGAL, it is evident that any NGAL systematically released from malignantly transformed cells would be freely filtered by the kidney glomerulus, however, may be expected to be largely reabsorbed by efficient endocytic mechanisms in the proximal tubules. Therefore, urinary excretion of NGAL is more likely to be present in tandem with a concomitant renal tubular injury that increases *de novo* NGAL synthesis and/or precludes NGAL reabsorption. We demonstrated that, in ccRCC patients, the mean value of urinary NGAL is higher than that observed in the urine of the control group. Our data support the findings of Morrisssey *et al*(20). Meanwhile, like Morrissey *et al*, we identified no correlation with tumor size or stage. With regard to MMP-9/NGAL complex, recent evidence suggests that urinary detection of the complex may represent a new biomarker for the prediction of cancer disease (21–23). In particular, MMP-9/NGAL enzymatic activity was observed in the urine of breast cancer patients but not in healthy controls (21), and in 50% of urine samples from prostate cancer patients and in 49% of the urine from bladder cancer patients (22). Evaluation of MMP-9 and MMP-9/NGAL complex in urine of patients with brain tumors revealed significantly higher expression levels compared with controls (23), which was also confirmed in tumor tissue. Following tumor resection, clearing of biomarkers was observed. These findings have led to the suggestion that the urinary detection of MMP-9/NGAL complex may represent a novel biomarker with potential for generalized application in cancer diagnostics and prognostics. However, no studies of urinary MMP-9/NGAL complex in kidney carcinoma are currently available. By ELISA, we detected no MMP-9/NGAL in urine specimens of oncocytoma and ccRCC patients and only in 10% of urine specimens from healthy individuals. The source of the MMP-9/NGAL complex in urine remains unknown. In fact, due to its large size, it seems unlikely that the MMP-9/NGAL complex is capable of being directly filtered from the serum to the urine. Instead, it is likely that MMP-9 and NGAL are mainly secreted into the blood by neutrophils infiltrating the tumors and are separately excreted in urine where they subsequently from the complex (6).

In spite of recent evidence and interest among biologists and oncologists, the serum and urine levels of MMP-9, NGAL and their complex (MMP-9/NGAL) appear to not provide an adequate test to identify kidney cancer. Nevertheless, due to the small number of patients included in the studies, the conclusion may not be transferable to the general population and therefore need further evaluation for validating diagnostic and prognostic utility.

## Figures and Tables

**Table I:** Serum levels of MMP-9, NGAL and MMP-9/NGAL in oncocytoma patients.

Case no.	Age (years)	Gender	Stage	Grade	NGAL (ng/ml)	MMP-9 (ng/ml)	MMP-9/NGAL (ng/ml)
P1	42	F	T1 N0 M0	G1	11	259	145
P2	66	M	T1 N0 M0	G1	26	142	71
P3	59	F	T2 N0 M0	G1	16	82	18
P4	59	F	T1 N0 M0	G1	22	327	85

NGAL, neutrophil gelatinase-associated lipocalin; MMP-9, matrix metalloproteinase 9.

**Table II:** Serum levels of MMP-9, NGAL and MMP-9/NGAL in clear cell renal cell carcinoma patients.

Case no.	Age (years)	Gender	Stage	Grade	NGAL (ng/ml)	MMP-9 (ng/ml)	MMP-9/NGAL (ng/ml)
P5	69	M	T1 N0 M0	G1	31	168	56
P6	54	M	T1 N0 M0	G1	54	355	114
P7	53	M	T1 N0 M0	G2	750	173	62
P8	60	F	T1 N0 M0	G2	306	609	42
P9	51	F	T1 N0 M0	G2	39	356	160
P10	63	F	T1 N0 M0	G2	18	428	92
P11	63	M	T2 N0 M0	G2	67	312	102
P12	60	F	T2 N0 M0	G2	37	575	237
P13	40	M	T2 N0 M0	G3	32	291	84
P14	73	M	T2 N0 M0	G3	80	202	14
P15	70	M	T2 N0 M0	G3	6	497	117
P16	61	M	T2 N0 M0	G3	58	681	306
P17	73	F	T2 N0 M0	G3	70	730	229
P18	43	M	T2 N0 M0	G3	60	499	177
P19	67	M	T3 N0 M1	G3	15	309	69
P20	57	F	T3b N0 M1	G3	18	393	90

NGAL, neutrophil gelatinase-associated lipocalin; MMP-9, matrix metalloproteinase 9.

**Table III:** Urine levels of MMP-9, NGAL and MMP-9/NGAL in oncocytoma patients.

Case no.	Age (years)	Gender	Stage	Grade	NGAL (ng/ml)	MMP-9 (ng/ml)	MMP-9/NGAL (ng/ml)
P1	42	F	T1 N0 M0	G1	4.7	8.13	N.D.
P2	66	M	T1 N0 M0	G1	0.1	N.D.	N.D.
P3	59	F	T2 N0 M0	G1	3.2	N.D.	N.D.
P4	59	F	T1 N0 M0	G1	0.1	N.D.	N.D.

N.D., none detectable; NGAL, neutrophil gelatinase-associated lipocalin; MMP-9, matrix metalloproteinase 9.

**Table IV:** Urine levels of MMP-9, NGAL and MMP-9/NGAL in clear cell renal cell carcinoma patients.

Case no.	Age (years)	Gender	Stage	Grade	NGAL (ng/ml)	MMP-9 (ng/ml)	MMP-9/NGAL (ng/ml)
P5	69	M	T1 N0 M0	G1	34.1	6.93	N.D.
P6	54	M	T1 N0 M0	G1	20.2	N.D.	N.D.
P7	53	M	T1 N0 M0	G2	116	0.55	N.D.
P9	51	F	T1 N0 M0	G2	41.1	N.D.	N.D.
P13	40	M	T2 N0 M0	G3	0.9	1.68	N.D.
P14	73	M	T2 N0 M0	G3	26.5	N.D.	N.D.
P15	70	M	T2 N0 M0	G3	34.4	22.80	N.D.
P19	67	M	T3 N0 M1	G3	3.8	6.13	N.D.
P20	57	F	T3b N0 M1	G3	1.9	1.35	N.D.

N.D., none detectable; NGAL, neutrophil gelatinase-associated lipocalin; MMP-9, matrix metalloproteinase 9.
